# Clinical efficacy of weight loss herbal intervention therapy and lifestyle modifications on obesity and its association with distinct gut microbiome: A randomized double-blind phase 2 study

**DOI:** 10.3389/fendo.2023.1054674

**Published:** 2023-03-22

**Authors:** Ming-Zhuo Cao, Chun-Hua Wei, Ming-Chun Wen, Ying Song, Kamal Srivastava, Nan Yang, Yan-Mei Shi, Mingsan Miao, Danna Chung, Xiu-Min Li

**Affiliations:** ^1^ Academy of Chinese Medical Sciences, Henan University of Chinese Medicine, Zhengzhou, China; ^2^ Department of Medicine, Wei-en Hospital, Weifang, China; ^3^ Department of Pathology, Microbiology & Immunology, New York Medical College, Valhalla, NY, United States; ^4^ General Nutraceutical Technology, LLC, Elmsford, NY, United States; ^5^ Healthy Freedom LLC, King of Prussia, PA, United States

**Keywords:** “W-LHIT” capsule, obesity, weight loss, gut microbiome, *Akkermansia muciniphila*, lifestyle modifications

## Abstract

**Goals:**

To assess the efficacy and safety of Chinese Medicine Prescription “W-LHIT” in subjects with simple obesity, and to explore its potential mechanism of action.

**Methods:**

Thirty-seven patients aged 18 to 60 from Wei-En hospital (Weifang City, Shandong, China), participated in a double blinded, placebo-controlled study. Subjects were randomly divided into 2 groups, 18 in treatment and 19 in placebo group. The treatment group took the “W-LHIT” capsules for two months, while the control group received placebo capsules. Both groups accepted healthy lifestyle education materials. After a 2-month treatment, the placebo group transferred to open-label treatment after unblinding.

**Results:**

72.22% participants in the treatment group lost more than 5% of their body weight, compared with 36.84% in the placebo group (*p* < 0.001). Body weight loss and body mass index reduction of the treatment group were also significantly higher than those of the placebo group (*p* < 0.05). These changes were accompanied by increased abundance of *Akkermansia muciniphila* and *Enterococcus faecium*, and decreased abundance of *Proteobacteria* in gut microbiota. Furthermore, the treatment group also showed improvement in obesity-related comorbidities such as hypertension and elevation of liver enzymes. No serious adverse reactions were found during the study period. Weight did not rebound at a follow-up visit 2 months after treatment.

**Conclusion:**

W-LHIT significantly improved body weight and comorbid conditions without obvious adverse reaction or rebound weight gain. These effects were associated with increased abundance of probiotics in gut microbiota. W-LHIT may have a potential for treating obesity in conjunction with healthy lifestyle modifications.

## Introduction

Obesity has been increasing in prevalence worldwide and is a serious threat to public health. Over the past 30 years, the prevalence of obesity among adults has exceeded 50% in some western developed countries (1). This increase is also observed in non-western and some developing countries, especially in China, where the number of overweight and obese people has been increasing at an alarming rate (2). A surge of related chronic diseases is strongly associated with the rising rate in obesity (3), such as type 2 diabetes, hypertension, atherosclerosis, cancers, and stroke, all of which have been major health threats in China for the past two decades (2). During the ongoing COVID-19 pandemic, obesity was found in early studies (4, 5) to be the second strongest predictor of hospitalization and need for mechanical ventilation in the elderly. In obese individuals, there is a reduced quality of life and decline in life expectancy (1). Due to increasing awareness of the risks of obesity, interest in weight loss management has grown significantly with calls for effective interventions.

Unhealthy eating styles and sedentary behavior and physical inactivity have been viewed as the key factors that contribute to obesity. Current guidelines recommend diet, exercise, and behavior modification as standard treatments for obesity. However, a study found that only 20% of adults who either tried to lose weight or to maintain their weight, could both eat fewer calories and complete at least 150 minutes of exercise per week (6). Simple exercise and diet are often challenging for patients attempting to maintain lasting results (7). Due to the risk of serious complications and the issues of nutritional management and treatment follow-up, bariatric surgery is only applicable to a specific subset of patients, and not widely applicable for clinical use (8). Pharmacological intervention can be an effective and widely accepted auxiliary method.

Weight loss herbal intervention therapy (W-LHIT) is a Chinese medicine (CM) prescription, consisting of 5 Chinese herbal medicines: *Ganoderma lucidum*, *Coptis chinensis*, *Astragalus membranaceus*, *Nelumbo nucifera gaertn* and *Fructus aurantii.* In our previous studies, we have showed that W-LHIT significantly and safely reduced the body weight of mice with high-fat diet, and normalized their glucose and cholesterol levels, without suppressing appetite (9). In addition, in our phase I clinical study with 14 patients with simple obesity, the W-LHIT capsules demonstrated clinically significant weight loss (Cao et al. Manuscript submitted).

Increasing evidence suggested gut microbiota played a significant role in the development of obesity. Many clinical or pre-clinical studies have shown that there are significant differences in the composition of gut microbiota between healthy versus obese individuals (10), with decreased diversity and richness in the gut microbiome of obese individuals (11). Also, causality between gut microbiota imbalance and obesity has been demonstrated in animal models. Gut microbiota dysbiosis can lead to an increase in lipopolysaccharide (LPS) in the host’s circulatory system and induce chronic and low-level inflammation, thereby playing an important role in the occurrence and development of metabolic diseases such as obesity (12–14). Recently, multiple studies have confirmed that traditional Chinese medicine can improve obesity in animal models by regulating the composition of gut microbiota (13, 15). Chih-Jung Chang et al. reported that water extract of *Ganoderma lucidum* could reduce obesity and inflammation in high fat diet (HFD) fed mice, which was associated with reversing HFD-induced gut dysbiosis (as indicated by the reduction of *Firmicutes*-to-*Bacteroidetes* ratios and endotoxin bearing *Proteobacteria* levels) and maintaining intestinal barrier integrity (16). *Fructus Aurantii* extract also showed a similar anti-obesity effect by modulating the gut microbiota (17). Berberine, the principal component of *Coptis chinensis*, is known as a potent anti-obesity and lipid lowering agent. Moreover, its anti-obesity effect could be associated with the increased butyrate production in the gut and modulation of the gut microbiota (18).

In this study, we aimed to further evaluate the safety and weight loss efficacy of the W-LHIT capsules, in combination with a standardized lifestyle intervention. In addition, we used 16S pyrosequencing technology to evaluate the shift in the composition of the gut microbiota before and after W-LHIT capsule treatment to explore its potential role in understanding W-LHIT capsules in weight loss response.

## Methods

### Participants and W-LHIT capsules preparation

Participants who met simple obesity criteria were recruited from the Weifang community through WeChat Friends Circle, advertisement, and local websites. Inclusion criteria were as follows: age 18-60 years with a body-mass index (BMI) of 28 - 48 kg/m^2^, stable body weight within the past 3 months, ability to engage in physical activity and follow healthy diet guidelines, no cardiovascular, hepatic, or renal disease. Patients with disease-induced obesity (such as Cushing’s syndrome or thyroid disease) were excluded. Exclusion criteria included the above-described diseases, psychiatric illness and psychological disorders, pregnancy and participants who cannot be clinically observed. Written informed consent was obtained from all subjects upon enrollment. The study was approved by the Medical Ethics Committee of Weifang Wei-En Hospital.

W-LHIT capsules were prepared in a GMP facility (Tian-jiang Pharmaceutical, Jiangsu, China). In our early research, we established HPLC fingerprints of individual herbal components, and monitored the quality of different batches of W-LHIT products by comparing the peak times and intensities of the identified compounds. Berberine was used as a key compound index (9). Nine capsules are equivalent to the daily crude herbal medicines dosage for 75 kg individual. The placebo capsule was filled with starch with the same weight and color as the formula.

### Study design

Before enrollment, a CT scan of the chest was used to exclude lung disease and a color doppler ultrasound was used to exclude organic diseases of the heart, liver, and kidneys. Thyroid function, blood coagulation, and sex hormone lab tests were used to rule out obesity caused by endogenous diseases.

The design of the study was a randomized double-blind placebo-controlled trial. Fourty participants were randomly divided into treatment group (treated with W-LHIT capsules) and control group (treated with placebo) according to random number table with 20 cases in each group. All participants had a light diet and were advised to drink at least 1.5 L of water during the study. W-LHIT was dosed according to body weight, all subjects dosed 9 to 15 capsules daily (3 - 5 capsules a time, before meals, three times a day) for 2 months and then followed up for an additional 2 months. Other weight-loss drugs (including weight-loss dietary supplements) were discontinued during the treatment and follow up period. If a subject discontinued the study prematurely, the remainder of doses were recalled. Since researchers and subjects were both blinded, W-LHIT capsules and placebo were all distributed by a third-party designated distributor. After unblinding, the subjects in control group voluntarily participated in another open trial where they were given a 2-month supply of W-LHIT capsules for treatment, while the active group stopped W-LHIT. Both groups had their weight checked again 2 months later.

### Primary outcomes

The primary core measurements included the changes in body weight (kg), BMI (kg/m^2^), hip circumference (cm), waist circumference (cm), blood pressure (Seat systolic blood pressure, SSBP; seat diastolic blood pressure, SDBP, mmHg) and heart rate (sub/min), and they were assessed at the beginning (0 M, one day before treatment), mid-term (1 M, 30 days after treatment), and endpoint (2 M, 60 days after treatment) of the weight loss intervention (please see the supporting information for detailed methods). The proportion of individuals losing more than 5% of baseline weight was also assessed at the end of the intervention, including the open trial.

### Secondary outcomes

The Secondary measurements included assessments of fasting blood glucose, high-sensitivity C-reactive protein (hs-CRP), liver function including alanine aminotransferase (ALT), aspartic aminotransferase (AST), glutamate transferase (GGT), total bilirubin (TBIL), total protein (TP) and albumin (A), renal function including urea nitrogen (BUN), and creatinine (CRE), highly sensitive C - reactive protein (CRP), fasting lipids including cholesterol (TC), triglycerides (TG), low-density lipoprotein (LDL-C) and high-density lipoprotein cholesterol (HDL-C), oral glucose tolerance test (OGTT; 75-g glucose, glucose, insulin, and C-peptide concentration changes from 0 to 120 min), and analysis of human fat composition, bone mineral density, bilateral knee joint, and lumbar vertebrae X-ray.

### Healthy lifestyle interventions

Healthy lifestyle interventions were carried out throughout treatment. All subjects were provided personalized healthy diet guidance according to their living habits, physical condition, and work characteristics. They were also encouraged to adhere to or increase physical exercise based on their physical examination results and their physical condition. However, there was not an obligatory requirement to engage in a strict diet or exercise program. All subjects regularly attended classes on the risk of obesity and how to develop a healthy lifestyle. A WeChat group was established to supervise all enrolled subjects. A contracted nurse reminded all the subjects to take the medication every day, to follow diet and exercise recommendations, and to post recipes for meals. Subjects were able to report their diet and exercise every day and share their weight loss symptoms and experience in this group forum.

### Safety assessment

Safety assessment included adverse events and standard laboratory tests (hematological and biochemical tests). Adverse events were recorded by the nurse through the WeChat group. A physical examination and an exercise cardiopulmonary function test (pulmonary function, electrocardiogram, and finger oxygen %) were performed at enrollment and on the 60th day.

### 16S Pacbio sequencing of fecal sample DNA

Fresh fecal samples were collected before and after the treatment for the gut microbial analysis. DNA was extracted from the subjects’ feces using a Power Soil^®^ DNA Isolation kit (MO BIO Laboratories, Inc., Carlsbad, CA, USA) according to the manufacturer’s instructions. The bacterial 16S rDNA was amplified by PCR using universal primers (27F 5′ -AGRGTTTGATYNTGGCTCAG-3′, 1492R 5′ -TASGGHTACCTTGTTASGACTT-3′). 16S Pacbio sequencing of the PCR products was performed on an Illumina MiSeq platform at Biomarker Technologies Co, Ltd. (Beijing, China).

After using the SMRT Link tool (version 8.0, provided by Pacbio), then Lima v1.7.0 software to obtain the Barcode-CCS sequence data, UCHIME v8.1 software to remove the chimera sequence, the optimization-CCS sequence was obtained for bioinformatics analysis. Trimmed sequences from each sample were clustered into operational taxonomic units (OTUs) based on a 97% sequence similarity using USEARCH v10.0 method. The taxonomical analysis was performed by alignment with the Bacterial Silva database (Release132, http://www.arb-silva.de). Alpha diversity indexes were evaluated based on the richness (Chao 1, Ace) and diversity (Shannon index, Simpson index) of these OTUs using Mothur v.1.30 (http://www.mothur.org/). A PLS-DA analysis (Partial Least Squares Discriminant Analysis) was performed according to the supervised matrix of distance. The linear discriminant analysis effect size (LEfSe) was conducted for the quantitative analysis of biomarkers (LDA threshold > 4) among each group. A Metastats analysis was used to identify the most differently abundant taxa between the placebo and treatment groups. A predicted KEGG pathway was also performed using Picrust software to obtain significant differences in gene function of the flora in the placebo and treatment groups.

### Statistical analysis

Data were exhibited as the mean with [95% confidence interval] (CI),95(n=18 or 19). All statistical analyses were performed using one-way ANOVA followed by Bonferroni *post hoc* by Prism 9 software (GraphPad Software, Inc, La Jolla, CA). P value smaller than 0.05 considered statistically significant.

## Results

### Weight loss

14 women and 26 men who met the screening criteria were recruited. 2 participants withdrew due to personal issues, 1 participant withdrew due to treatment compliance issues, and 37 participants (active n=18 vs placebo n=19) completed the trial. As shown in **Table 1**, baseline characteristics at randomization were compared between the treatment group and placebo group.

**Table 1:** Baseline data.

	Treatment (n=18)	Control (n=19)	P value
Sex (F/M)	6/12	7/12	0.93
Age (y)	39 ± 6.4	39 ± 4.6	0.92
Body Weight (kg)	96 ± 7.5	95 ± 8.7	0.92
BMI	32.25 ± 1.4	34.04 ± 2.5	0.21
Hip Circumference (cm)	115 ± 2.5	116 ± 4.8	0.51
Waist Circumference (cm)	107 ± 4.8	108 ± 6.0	0.78
SDBP (mmHg)	91 ± 5.9	90 ± 5.2	0.98
SSBP (mmHg)	135 ± 9.5	133 ± 8.7	0.76
HR	77 ± 4.6	78 ± 4.0	0.68
HS-CRP (mg/L)	4.5 ± 1.4	4.9 ± 1.5	0.87
Total cholesterol (mmol/l)	5.4 ± 0.5	5.2 ± 0.9	0.80
Triglycerides (mmol/l)	2.0 ± 0.6	3.0 ± 1.1	0.16
HDL cholesterol (mmol/l)	1.1 ± 0.08	1.2 ± 0.15	0.24
LDL cholesterol (mmol/l)	3.3 ± 0.3	3.1 ± 0.4	0.68

F, female; M, male; y, year; BMI, body mass index; SDBP, sitting diastolic blood pressure; SSBP, sitting systolic blood pressure; HR, heart rate, beats per minute; HDL, high-density lipoprotein; LDL, low-density lipoprotein. Data shown as mean with (±) 95% CI.

**Figure 1:**
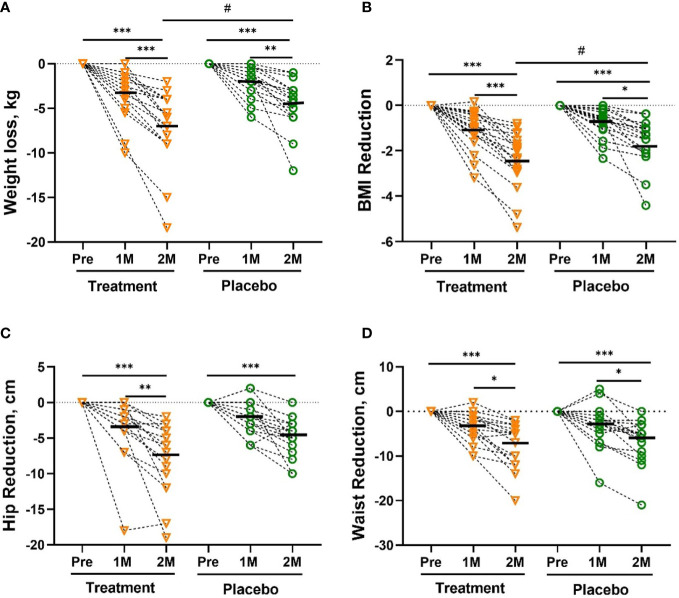
The Reduction in body weight **(A)**, BMI **(B)**, hip circumference **(C)**, waist circumference **(D)**. The reduction in body weight was significantly greater in the treatment group than in the placebo group after 2 months (2M). Bars were shown as mean of each group. All intra-group analysis were performed by using one-way ANOVA followed by bonferroni *post hoc*, and all inter-group analysis for baseline and treatment effects were performed by using t test followed by Mann-Whitney (# represents *p* < 0.05) by Prism 9 software (*, ** and *** represent *p* < 0.05, *p* < 0.01 and *p* < 0.001). Body mass index, BMI.

Weight loss during the study is shown in **Figure 1A**. From randomization to the 60th day (2 months), both groups showed sustained weight loss, but mean weight loss and BMI reduction of W-LHIT capsule-treated subjects were significantly greater than that of placebo group, with mean weight reduction of -7.05 [-9.10, -4.99] kg (- 7.37%, *p <*0.001) in treatment group, compared to -4.39 [-5.68, -3.09] kg (- 4.78%, *p <*0.001) in placebo group (*p <*0.05, **Figure 1A**). Similar results were obtained for BMI, with significant reduction in both groups. The mean BMI in the treatment group decreased by -2.39 [-3.00, -1.77] kg/m^2^ (- 7.40%, *p <*0.001), significantly higher than the reduction of -1.61 [-2.09, -1.12] kg/m^2^ (- 4.73%, *p <*0.001) in the control group (*p <*0.05, **Figure 1B**). Both groups showed a reduced hip circumference (-7.61cm verse -4.32 cm, *p* = 0.067, **Figure 1C**) and waist circumference (-7.44 cm verse -6.79 cm, *p* = 0.71, **Figure 1D**), but without statistically significant difference between the groups. During the study, 72.22% of the subjects in the treatment group lost more than 5% of their body weight and 77.78% of the subjects lost at least 5% of their BMI, much higher than the control group (36.84% both in body weight and BMI), as shown in **Table S1**. Encouragingly, the number of participants in the treatment group whose BMI decreased from obesity to overweight (5 verse 3) and from severe obesity to obesity (5 verse 3) was much higher than that in the control group.

The results of the analysis of human fat components were consistent with the results of the above weight loss. Body fat rate, fat weight, fat-free body weight in both groups were significantly reduced. However, the percentage of skeletal muscle was only significantly increased in the treatment group. Moreover, the reduction of body fat rate (- 14.8% vs - 4.56%, *p* < 0.01), body fat (- 10.0% vs - 3.6%, *p* < 0.01) and fat-to-muscle ratio (- 12.0% vs - 3.33%, *p* < 0.01) in treatment group was statistically significant compared with that of the placebo group (**Table S2**).

### Changes in blood pressure, blood sugar, and blood lipid

**Figure 2:**
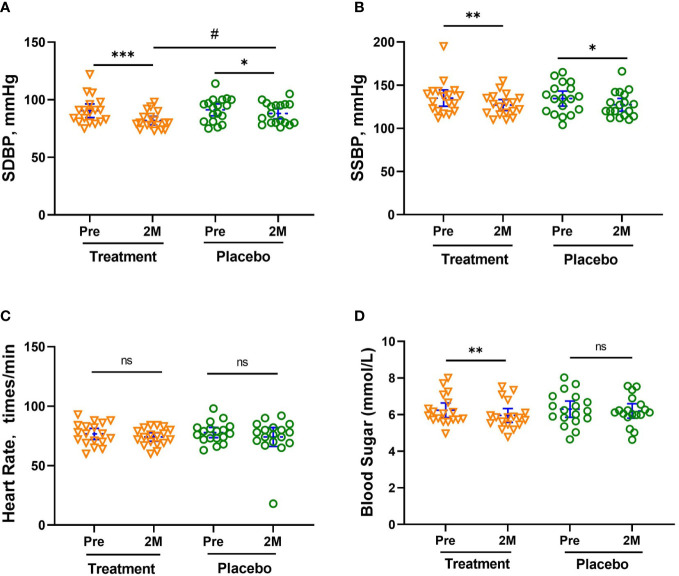
Mean Changes in SDBP **(A)**, SSBP **(B)**, HR **(C)** and Blood sugar levels **(D)**. The reduction in SDBP was significantly greater in the treatment group than in the placebo group after 2 months (2M). Bars (blue) were shown as mean of each group with 95% CI. All intra-group analyses were performed using one-way ANOVA followed by Bonferroni *post hoc*, and inter-group was performed by using t test followed by Mann-Whitney by Prism 9 software, (*, ** and *** represent *p* < 0.05, *p* < 0.01 and *p* < 0.001; # represents p < 0.05, ns represents no significant difference). Seated systolic blood pressure, SSBP, seated diastolic blood pressure, SDBP, mmHg.

As shown in **Figures 2A, B**, the blood pressure of both groups decreased significantly. The seated diastolic blood pressure (SDBP) decreased by -8.67 [-12.59, -4.64] mmHg (- 9.52%, *p <*0.001) and -3.21 [-6.47, -0.083] mmHg (- 3.59%, *p <*0.05), respectively, with a significant difference between the two groups (*p <*0.05, **Figure 2A**). The seated systolic blood pressure (SSBP) decreased by -7.99 [-13.29, -2.598] mmHg (- 5.88%, *p <*0.05) and -7.16 [-13.67, -0.6621] mmHg (- 5.33%, *p <*0.05), in the treatment and placebo groups, respectively. Among the subjects, there were 13 people with hypertension, 6 people in the treatment group (5 people at Stage 1 hypertension, 1 person at Stage 2), and 7 people in the placebo group (6 people at Stage 1, 1 person at Stage 2). There were 5 subjects in the treatment group and 2 subjects in the placebo group whose blood pressure returned to normal after 2 months of treatment. There was no noticeable change in heart rate for all subjects (**Figure 2C**).

The fasting blood sugar level of subjects in the treatment group also showed a significant reduction of -0.28 [-0.46, -0.096] mmol/L, *p* < 0.01), while that of subjects in the placebo group only decreased slightly (- 0.1 [-0.17, 0.095] mmol/L, shown in **Figure 2D**). All subjects in the treatment group whose blood sugar level exceeded the threshold (6.1 mmol/L) had varying degrees of blood glucose reduction. The blood sugar of the 4 subjects with blood glucose levels between 6.1-6.7 mmol/L fell below the threshold. In addition, all the concentration of glucose tolerance, C-peptide, and insulin in the treatment group significantly decreased after W-LHIT treatment, and their mean area under the curve (AUC) decreased by 5.87%, 10.54% and 32.99% respectively. While the fasting blood glucose and the above indicators in placebo group declined at individual time points, there was no significant pattern of change at most time points (**Table 2**).

**Table 2:** Results of GGT, CRT and ICT in the two group before and after treatment.

Characteristic		Normal values	Treatment (n=18)		Placebo (n=19)	
			Baseline	Post-treatment	Baseline	Post-treatment
Glucose (mmol/L)	Fasting	3.90-6.10	5.67 ± 0.8	5.50 ± 0.68	5.58 ± 0.81	5.53 ± 0.77
	30 min.	7.78-8.89	9.55 ±1.29	8.98 ± 1.13***	8.82 ± 1.47	8.58 ± 1.45*
	60 min.	7.78-8.89	9.47 ± 2.87	8.96 ± 2.53*	8.32 ± 2.99	8.55 ± 2.91
	120 min.	3.90-7.80	7.09 ± 1.59	6.60 ± 1.9**	7.18 ± 2.21	7.03 ± 2.03
	180 min.	3.90-6.10	5.2 ± 1.73	4.89 ± 1.38*	5.00 ± 1.17	5.38 ± 1.35
	AUC		1379 ± 135.8	1298 ± 130.6	1317 ± 151.7	1324 ± 145.2
C-peptide (ng/mL)	Fasting	0.52--4.38	3.72 ± 1.27	3.22 ± 0.86*	3.96 ± 1.37	3.86 ± 1.29
	30 min.	3.58--13.2	11.16 ± 4.48	9.89 ± 3.68**	9.5 ± 3.37	9.86 ± 3.7
	60 min.	3.58--13.2	12.49 ± 4.05	11.13 ± 2.48*	10.02 ± 2.74	11.24 ± 2.76
	120 min.	1.2--11.3	10.66 ± 3.63	9.46 ± 2.68**	10.24 ± 3.13	10.22 ± 2.54
	180 min.	0.38--6.56	6.34 ± 2.76	6.05 ± 2.28*	6.08 ± 2.6	6.75 ± 2.76
	AUC		1783 ± 248.6	1595 ± 188.4	1676 ± 188.2	1592 ± 199.4
Insulin (μU/mL)	Fasting	2.3--26.0	21.44 ± 11.01	17.57 ± 7.689*	24.09 ± 14.19	21.99 ± 12.26
	30 min.	10.5--61.8	170.33 ± 125.32	126.39 ± 72.06**	120.6 ± 84.15	108.66 ± 74.98*
	60 min.	10.5--61.8	155.62 ± 95.62	92.31 ± 55.29***	104.76 ± 57.01	122.09 ± 62.14
	120 min.	1.02-- 41.09	112.34 ± 74.74	71.06 ± 39.55**	104.08 ± 63.73	88.59 ± 43.48
	180 min.	0.25-- 11.53	41.55 ± 33.24	32.03 ± 23.13*	34.98 ± 35.88	40.85 ± 40.40
	AUC		20422 ± 5487	13684 ± 3112	15999 ± 3583	15863 ± 4092

Data are shown as mean ± SD. Intra-group analyses were performed by using t test followed by Wilcoxon by Prism 9 software, p value, **< 0.01, ***< 0.001. GTT, Glucose tolerance test; CPRT, C-peptide release test; IRT, Insulin release test; F.B.S, Fasting blood sugar.

**Figure 3:**
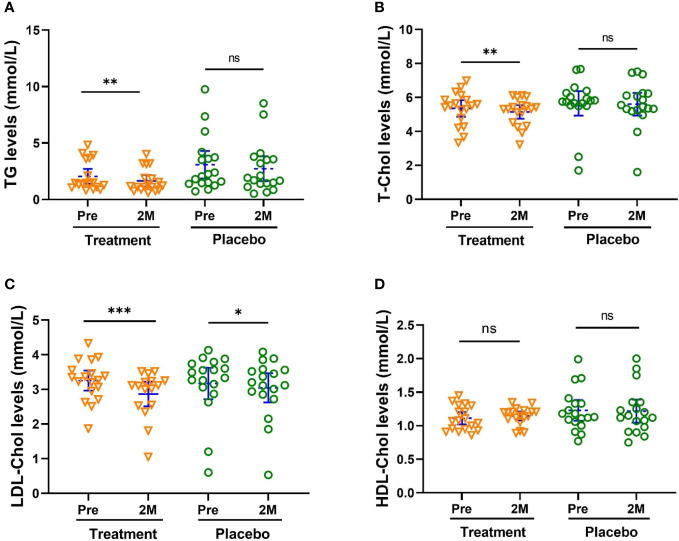
W-LHIT significantly reduced total cholesterol (TC), blood triglycerides (TG) and low-density lipoprotein cholesterol (LDL-C). **(A)** TC; **(B)** TG; **(C)** LDL-C; **(D)** HDL-C. Bars were shown as mean of each group with 95% CI. All intra-group analyses were performed using one-way ANOVA followed by Bonferroni *post hoc*, and inter-group was performed by using t test followed by Mann-Whitney by Prism 9 software (*, ** and *** represent *p* < 0.05, *p* < 0.01, *p* < 0.001, ns represents no significant difference). TC, Total cholesterol; TG, triglycerides; LDL-C, low-density lipoprotein; and HDL-C, high-density lipoprotein cholesterol.

Additionally, W-LHIT significantly reduced total cholesterol (TC, **Figure 3A**, *p* < 0.01), blood triglycerides (TG, **Figure 3B**, *p* < 0.01) and low-density lipoprotein cholesterol (LDL-C, **Figure 3C**, *p* < 0.001). The concentrations of TC, TG and LDL-Chol in the treatment group were decreased by 3.87%, 19.2% and 12.0%, respectively. There were no noticeable changes in TC, TG, HLD-C and for the placebo group (**Figure 3**).

**Figure 4:**
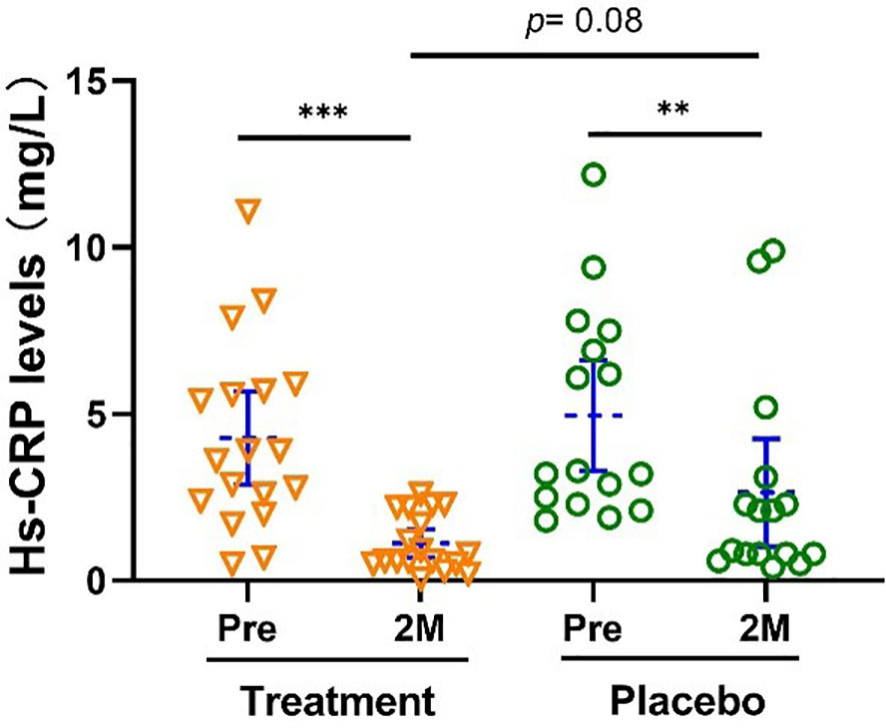
Hs-CRP level was significantly reduced after the treatment. Bars were shown as mean of each group with 95% CI. Intra-group analyses were performed using one-way ANOVA followed by Bonferroni *post hoc*, inter-group analyses were performed by using t test followed by Mann-Whitney by Prism 9 software (** and *** represent *p* < 0.01 and *p* < 0.001). Hs-CRP, High-sensitivity C-reactive protein.

Hs-CRP is positively correlated with atherosclerosis. Subjects in both treatment and control groups also showed a significant reduction in their Hs-CRP level, (-73.73% [-60.71%, -83.24%], *p <*0.001 vs -46.77% [-14.04%, - 79.51%], *p <*0.01, **Figure 4**).

### Effect on body weight 2 months off double blind placebo-controlled trialand open trial

**Figure 5:**
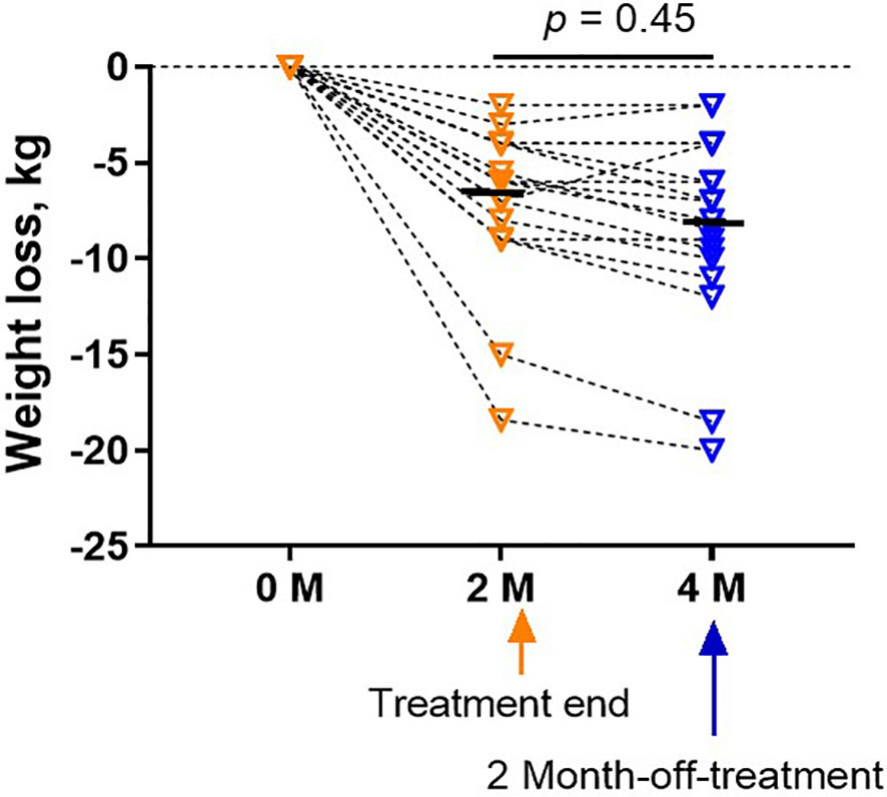
Weight did not rebound in the treatment group at a follow-up visit 2 months after the treatment. Bars were shown as mean of each group. The analyses were performed using one-way ANOVA followed by Bonferroni *post hoc* by Prism 9 software.

Two months after the end of treatment, the body weight of all subjects in the treatment group maintained at the same level after treatment or slightly decreased (*p* = 0.45, **Figure 5**) for the following two months after completion of therapy.

After unblinding, 12 of the 19 subjects in the placebo group subsequently completed an additional two months of W-LHIT capsules treatment. As shown in **Figure S1**, in the absence of healthy lifestyle interventions, only one-third of subjects lost nearly 5% of their body weight, with a mean weight and BMI loss of 3.13 [4.23, 2.03] kg (-3.60%) and 1.13 [1.57, 0.70] kg/m^2^ (-3.51%). The reductions in systolic and diastolic blood pressure were consistent with those in the treatment group, by 2.49% and 9.80%, respectively (*p* < 0.001).

### Laboratory safety profile

Most subjects at enrollment had mild to moderate fatty liver, and 14 subjects had moderate fatty liver. During the treatment, 6 subjects (5 in the treatment group) with moderate fatty liver improved to mild fatty liver, and 4 subjects with mild fatty liver in the treatment group returned to normal. Among them in the treatment group whose glutamyl transferase (GGT, liver function index, **Figure S2**) levels was above the threshold, their GGT levels decreased significantly after treatment, while that of subjects in the placebo group showed no significant changes.

There was no statistically significant difference between the treatment group and the placebo group in the levels of other liver and kidney function indices, including alanine aminotransferase (ALT), aspartate aminotransferase (AST), total bilirubin (TBIL), total protein, and albumin. The electrolytes, routine urine, blood coagulation, and routine blood of all subjects were within the normal range, and there was no significant change (**Table S3**).

### Clinical adverse reactions

Seven subjects reported slight gastrointestinal (GI) reactions (including 5 subjects in the open trail, **Table S4**), no subjects reported obvious adverse symptoms during the whole W-LHIT treatment. The GI side effects included decreased hyper appetite, mild nausea, and increased frequency of stools. All subjects alleviated these side effects quickly by taking W-LHIT after meals or reducing the dose.

### Characteristics of 16S Pacbio sequencing results

**Figure 6:**
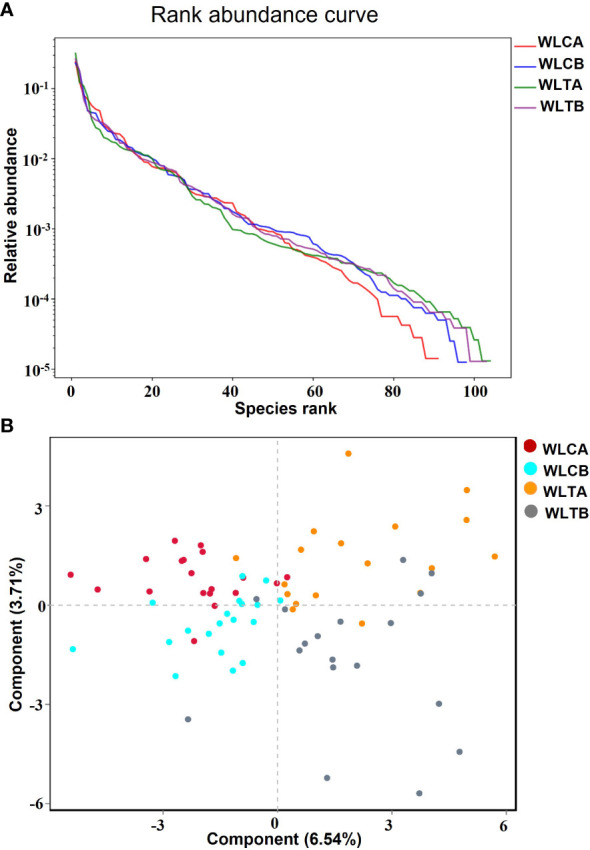
**(A)** Rank abundance curve. Each curve represents a group; placebo group (WLCB (baseline), WLCA (post treatment)) and treatment group (WLTB (baseline) and WLTA (post treatment)). **(B)** PLS-DA plots based on unweighted unifrac metrics. Each symbol represents a sample from the placebo group or treatment group, respectively.

The effects of W-LHIT on the intestinal microbiota composition were assessed by sequencing the bacterial 16S rRNA. A total of 417,913 optimization-CCS sequences and 352 OTUs (97% similarity) were obtained from the 74 samples through a single molecules real-time sequencing analysis, with an average of 5,647 reads and 82 OTUs per sample (**Table S5**). These reads/OTUs were assigned to 11 different phyla (15 class, 27 order, 52 family, 112 genus, and 219 species, and the principal bacterial phyla of all groups were *Firmicutes*, *Proteobacteria*, and *Bacteroides*. Main changes in microbial diversity observed before and after treatment included the enrichment of *Proteobacteria* (a slight increase (29.8% vs 32.8%) in the placebo group versus a slight decrease (32.3% vs 29.8%) in the treatment group), *Verrucomicrobiota* (a decrease (1.73% vs 0.7%) in the placebo group but an obvious increase (4.47% vs 10.52%) in the treatment group), and *Bacteroidetes* (a parallel decrease in both groups, 24.04% vs 20.86% in the placebo group and 22.75% vs 18.31% in the treatment group) (**Table S6** and **Figure 6B**) showed details of the composition on Genus in both group.

The alpha diversity indexes, including Ace and Chao indices, rarefaction, Shannon-index (**Table S4**), and rank abundance curve (**Figure 6A**), indicated that there was similar richness and sufficient sequence coverage in all samples. However, the placebo group had a significant decrease in reads, OUTs, and the classification of microorganisms (family, genus, species), while the treatment group had a slight increase in OUTs and the classification of microorganisms (phylum, class, order, family, genus, species).

### Differences in microbial structure between the W-LHIT treatment group and the placebo group

**Figure 7:**
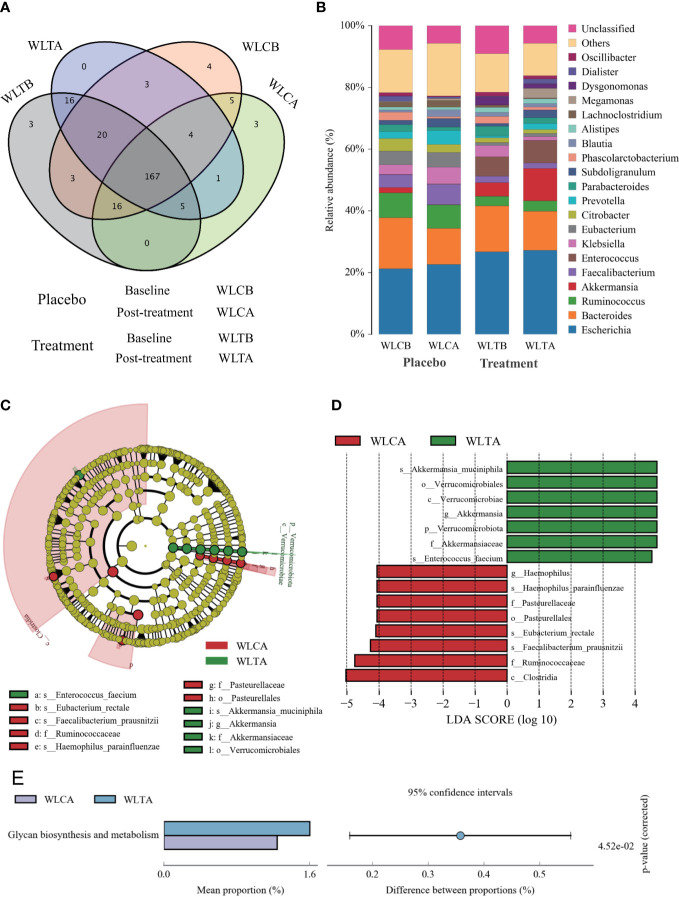
The structures and compositions of the gut microbiota before and after treatment in the two groups. **(A)** The Venn diagram of OTUs (species). **(B)** The composition of relative abundance on genus. **(C)** Phylogenetic cladogram of microbial lineage in fecal samples of treatment group and placebo group, with colors representing the most abundant differences in composition. **(D)** Key phylotypes of the gut microbiota responding to W-LHIT treatment. The histogram shows the lineage with LDA value of 4 or higher determined by LEFSe. **(E)** Key KEGG metabolic pathway responding to W-LHIT treatment.

To identify whether W-LHIT-mediated weight loss is associated with changes in the gut microbiota, we then profiled the overall microbial structure of the placebo and W-LHIT treatment groups. The PLS-DA results (**Figure 6B**) based on the weighted Unifrac distance matrix revealed that the overall structure of the bacteria did not change significantly in either group, and only a small part of the microbial structure between the two groups changed significantly (**Figures 7A, B** and **Tables S5, 6**). A LEfSe (Line Discriminant Analysis (LDA) Effect Size) analysis was used to discover the key biomarkers (LDA score > 4) of the gut microbiota under W-LHIT and placebo treatment, as shown in **Figures 7C, D**. The enriched phylotypes in the treatment group were the genera *Akkermansia* (*p* = 0.013, metastats) within the family *Akkermansiaceae* (*p*= 0.005, metastats) (within the order *Verrucomicrobiota* (*p* = 0.04, metastats), the species *Enterococcus_faecium* (*p* = 0.037, metastats) within the genera *Enterococcus* (*p* = 0.039, metastats). The enriched phylotypes in the placebo group were the species *Haemophilus_parainfluenzae* (*p* = 0.014, metastats) within the genera *Haemophilus* (*p* = 0.019, metastats), the species *Faecalibacterium_prausnitzii* (*p* = 0.006, metastats) and *Eubacterium_rectale* (*p* = 0.006, metastats) within the class *Clostridia* (*p* = 0.007, metastats). Furthermore, the analysis of the KEGG metabolic pathway found that only glycan biosynthesis and metabolism (**Figure 7E**) was statistically different between the treatment group and the placebo group.

## Discussion

In recent years, China has ranked first in the world for obesity and type 2 diabetes (19). Obesity related complications such as type 2 diabetes and hypertension have posed a serious threat to people’s health. There has been a growing consensus worldwide on the importance of obesity treatment not merely to achieve weight loss, but also to ameliorate adiposity-based complications (20, 21). Along with lifestyle intervention, W-LHIT capsule treatment resulted in 72.22% of subjects losing more than 5% of their body weight within 2 months, and 78.22% of the subjects to reduce more than 5% of their BMI. In addition, W-LHIT capsule can also reduce blood pressure, blood glucose and blood lipid of the subjects. In addition, W-LHIT capsule treatment also resulted in a greater reduction of blood pressure, blood sugar and blood fat. In this study, most of the subjects had impaired glucose tolerance, and about one third of the subjects were diabetic. In the treatment group, except for one diabetic subject whose blood glucose and blood lipid levels did not improve during the treatment period, other diabetic subjects showed significantly reduced levels of cholesterol, low-density lipoprotein, glucose tolerance, C-peptide release, and insulin release. In the placebo group, diabetic subjects showed little improvement in blood glucose and lipid levels. The results of subjects’ liver and kidney function, and other biochemical indicators indicate W-LHIT’s favorable safety and tolerance profile. These promising results indicate a potential role for W-LHIT for addressing weight control & management problems of simple obesity patients.

Unhealthy dietary habits and sedentary lifestyle are key contributing factors leading to obesity. Therefore, healthy lifestyle interventions (including healthy diet guidance and physical activity) can play a profound role in the treatment process. During the treatment, the average weight reduction of the placebo group was 4.58 kg (- 4.9%). For comparison, no healthy lifestyle intervention was performed in the open trial. The results showed that the body weight was only reduced by 3.6% in the open treatment trial, which was significantly lower than the average weight loss of the treatment group with lifestyle interventions (-7.05kg, -7.2%). Despite the insufficient sample size during open-treatment trials, we can still see the importance of healthy lifestyle interventions for obesity treatment. There was a limitation that the daily food intake calories and physical activity for each subject were not quantified during the treatment, and the healthy lifestyle intervention was tailored for each subject under the guidance of the physician. So, the difference in weight loss of subjects could have possibly been affected differently. The quality-of-life scale score did not measure changes of psychological and physiological functions before and after treatment. Improvement of the quality-of-life score could further contribute to enhancing weight loss (22). In subsequent research, further enhancement of the quality-of-life scale would be included.

As a marker of obesity, the level of hs-CRP is significantly elevated in the obese individual, and positively correlated with BMI obesity (23). Hs-CRP is synthesized by the liver in response to the stimulation of interleukin-6 and tumor necrosis factor-α, indicating a state of inflammation (24). In our study, the hs-CRP level decreased by 72.59% after W-LHIT treatment, which confirmed that managing obesity can help reduce the risk of cardiovascular disease and comorbidities by inhibiting the inflammatory mechanism (25).

Although there has been no consensus on how changes in the composition of gut microbiota can contribute to obesity, cumulative evidences have demonstrated that the occurrence of obesity is strongly associated with gut microbiota dysbiosis, which can result in chronic, persistent low-grade inflammatory reactions and abnormal lipid metabolism (12, 26, 27). In a review on the profile of the gut microbiota in obese adults, the consistent conclusion was that obese individuals (in comparison to leaner individuals) have a greater *Firmicutes/Bacteroidetes* ratio, *Fusobacteria*, *Proteobacteria*, *Mollicutes*, *Lactobacillus*, and reduced *Verrucomicrobia* (*Akkermansia muciniphila*), *Faecalibacterium* (*Prausnitzii*), *Bacteroides*, *Methanobrevibacter smithii*, *Lactobacillus plantarum (* 27). Our results support the trend that there is a negative correlation between Firmicutes/Bacteroidetes ratio and BMI. After W-LHIT treatment, the BMI of both groups decreased, but the *Firmicutes/Bacteroidetes* ratio both increased, with the mean ratio increased from 1.95 to 2.53 in the placebo group and from 1.54 to 1.78 in the treatment group. Compared with normal-weight individuals, obese individuals have a significant increase in *Proteobacteria* and a significant decrease in *Verrucomicrobia*. Shin et al. considered that an increased prevalence of *Proteobacteria* may be an active feature of metabolic disorders (28, 29). We found that the composition of *Proteobacteria* in 72.9% of the obese subjects exceeded 20% before treatment. The average composition of *Proteobacteria* in the treatment group decreased by about 2.6%, possibly indicating movement toward normalization of the gut microbiota, but in the placebo, group increased by about 3%. One of the new generation of probiotic candidates, *Verrucomicrobia* (*Akkermansia muciniphila*), can degrade mucin, and is closely related to host health (30). It has been found to enhance the intestinal barrier function and the effects of immunotherapy (31), enhance glucose tolerance and reduce insulin resistance (32), and moderate inflammatory responses (33, 34), exhibiting beneficial therapeutic roles in obesity, type 2 diabetes, atherosclerosis, tumors, and inflammatory bowel disease (IBD)- related gastrointestinal disturbances (30). Another exciting result in our study was that the abundance of *Verrucomicrobia* (*Akkermansia muciniphila*) in the W-LHIT treatment group significantly increased from 4.4% to 10.5%. Increasing evidence indicates that berberine (key compound index) target the gut microbiota and reversely modulate the structure and diversity under pathological conditions, thus exerting poly-pharmacological effects (18, 35) such as anti-obesity (36), anti-hyperlipidemia (37), anti-diabetes (38).

*Coptis chinensis* is the sovereign medicine in W-LHIT prescription. However, 2 subjects in the treatment group occasionally experienced mild gastrointestinal adverse events due to the large oral dose (9-15 capsules) of W-LHIT and the bitter taste of its main components (*Coptis chinensis*). *Coptis chinensis* has been used for thousand years in China safely at its therapeutic dose to treat various inflammatory disorders and related diseases, such as diarrhea, vomiting, abdominal distention, high fever coma, toothache, diabetes and eczema (39), and it is highly safe at its therapeutic dose. Linn et al. found 20 patients administered with *Coptis chinensis* at a daily dose of 3 g for 1055 patient-days without any organ toxicity or electrolyte imbalance (40). Our follow-up study will refine the ingredients further and enteric coating should be another good way to address this issue. *Coptis chinensis* is cold in nature (39), so, patients with weak spleen and stomach should use it with caution.

This study still has limitations. Firstly, no lean mass subjects were enrolled in this trial, the concern should be addressed in our follow-up study. Secondly, weight loss is a long-term and arduous work for subjects with sever obesity. So, only two months treatment is insufficient, and subjects with severe obesity need to receive longer intervention duration. Thirdly, this is a single-center study, limited by the small sample size, and some efficacy indicators between the treatment and control groups are not statistically significant, including the hip circumference (*p* =0.09) and waist circumference (*p* = 0.7). Due to the small sample size, several results of the two groups failed the normality test (alpha=0.05), so, the data processed by the 2-way ANOVA method shows that there is no statistical difference between them in weight loss, SDBP reduction and LDL-Chol reduction. The data processed by the unpaired t test followed by Mann-Whitney method have significant differences. In the follow-up study, we will further expand the sample size to obtain more meaningful results. Despite these limitations, we found that WLHT may be of great significance in managing weight loss of patients by ameliorating microbiome dysbiosis.

In conclusion, our current study assessed the efficacy and safety of W-LHIT capsules in 37 Chinese patients with simple obesity. We found that W-LHIT significantly reduced the weight of subjects with simple obesity, by greater than 5% of body weight in 72.6% of participants, which was significantly higher than in only 36.6% of the placebo group. In addition to weight loss, subjects in the treatment group also had significant improvements in blood pressure, blood glucose, and blood lipids. During the 2-month treatment, 7 subjects reported slight-mild gastrointestinal adverse reactions. However, with taking the medicine after meal or reducing the dosage, all the adverse reactions were gradually relieved. In addition, the results of 16S gut microbiota showed that W-LHIT significantly increased the abundance of *akkermansia muciniphila* and the *Firmicutes/Bacteroidetes* ratio, and decreased the abundance of *Proteobacteria*, facilitating the normalization of gut microbial ecosystem.

## Data availability statement

The raw data supporting the conclusions of this article will be made available by the authors, without undue reservation.

## Ethics statement

The studies involving human participants were reviewed and approved by Wei-En hospital. The patients/participants provided their written informed consent to participate in this study. Written informed consent was obtained from the individual(s) for the publication of any potentially identifiable images or data included in this article.

## Author contributions

C-HW, M-CW, and Y-MS were significantly involved in conducting experiments. M-ZC, YS, KS, and NY were involved in data analysis. M-ZC was significantly involved in manuscript preparation. X-ML, DC, and M-SM were significantly involved in study design, data interpretation, and manuscript revision. All authors contributed to the article and approved the submitted version. 

## References

[1] Ng MT Fleming M Robinson B Thomson N Graetz C Margono E. Global, regional, and national prevalence of overweight and obesity in children and adults during 1980–2013: A systematic analysis for the global burden of disease study 2013. Lancet (2014) 384(9945):766–81. doi: 10.1016/S0140-6736(14)60460-8 PMC4624264 24880830

[2] Zhang L Wang Z Wang X Chen Z Shao L Tian Y. Prevalence of overweight and obesity in China: Results from a cross-sectional study of 441 thousand adults, 2012-2015. Obes Res Clin Pract (2020) 14(2):119–26. doi: 10.1016/j.orcp.2020.02.005 32139330

[3] Knell G,Q Li K Pettee G Shuval K. Long-term weight loss and metabolic health in adults concerned with maintaining or losing weight: Findings from NHANES. Mayo Clin Proc (2018) 93(11):1611–6. doi: 10.1016/j.mayocp.2018.04.018 PMC6526934 30119916

[4] Michalakis K Ilias I. SARS-CoV-2 infection and obesity: Common inflammatory and metabolic aspects. Diabetes Metab Syndr (2020) 14(4):469–71. doi: 10.1016/j.dsx.2020.04.033 PMC7189186 32387864

[5] Belancic A Kresovic A Racki V. Potential pathophysiological mechanisms leading to increased COVID-19 susceptibility and severity in obesity. Obes Med (2020) 19:100259. doi: 10.1016/j.obmed.2020.100259 32501427 PMC7255205

[6] Maynard LM Serdula MK Galuska DA Gillespie C Mokdad AH. Secular trends in desired weight of adults. Int J Obes (Lond) (2006) 30(9):1375–81. doi: 10.1038/sj.ijo.0803297 16552407

[7] Bish CL Michels Blanck H Maynard LM Serdula MK Thompson NJ Kettel Khan L. Health-related quality of life and weight loss among overweight and obese u. s. adults, 2001 to 2002. Obes (Silver Spring) (2006) 14(11):2042–53. doi: 10.1038/oby.2006.239 17135622

[8] Turner M Johnson AC Lantz P. Impact of self-efficacy on risk aversion in the context of surgical weight loss decision scenarios. Clin Obes (2019) 9(4):e12311. doi: 10.1111/cob.12311 31050137

[9] Nan Yang DC Liu C Liang B Li X-M. Weight loss herbal intervention therapy (W-LHIT) a non-appetite suppressing natural product controls weight and lowers cholesterol and glucose levels in a murine model. BMC Complementary and Alternative Medicine (2014) 14(261). doi: 10.1186/1472-6882-14-261 PMC4125697 25055851

[10] Gao X Zhang M Xue J Huang J Zhuang R Zhou X. Body mass index differences in the gut microbiota are gender specific. Front Microbiol (2018) 9:1250. doi: 10.3389/fmicb.2018.01250 29988340 PMC6023965

[11] Le Chatelier E Nielsen T Qin J Prifti E Hildebrand F Falony G. Richness of human gut microbiome correlates with metabolic markers. Nature (2013) 500(7464):541–6. doi: 10.1038/nature12506 23985870

[12] Gomes AC Hoffmann C Mota JF. The human gut microbiota: Metabolism and perspective in obesity. Gut Microbes (2018) 9(4):308–25. doi: 10.1080/19490976.2018.1465157 PMC6219651 29667480

[13] Ding Y Song Z Li H Chang L Pan T Gu X. Honokiol ameliorates high-Fat-Diet-Induced obesity of different sexes of mice by modulating the composition of the gut microbiota. Front Immunol (2019) 10:2800. doi: 10.3389/fimmu.2019.02800 31921106 PMC6917612

[14] Fuke N Nagata N Suganuma H Ota T. Regulation of gut microbiota and metabolic endotoxemia with dietary factors. Nutrients (2019) 11(10):2277. doi: 10.3390/nu11102277 31547555 PMC6835897

[15] Hu J Huang H Che Y Ding C Zhang L Wang Y. Qingchang huashi formula attenuates DSS-induced colitis in mice by restoring gut microbiota-metabolism homeostasis and goblet cell function. J Ethnopharmacol (2021) 266:113394. doi: 10.1016/j.jep.2020.113394 32941971

[16] Chang CJ Lin CS Lu CC Martel J Ko YF Ojcius DM. Ganoderma lucidum reduces obesity in mice by modulating the composition of the gut microbiota. Nat Commun (2015) 6:7489. doi: 10.1038/ncomms8489 26102296 PMC4557287

[17] Bai YF Wang SW Wang XX Weng YY Fan XY Sheng H. The flavonoid-rich quzhou fructus aurantii extract modulates gut microbiota and prevents obesity in high-fat diet-fed mice. Nutr Diabetes (2019) 9(1):30. doi: 10.1038/s41387-019-0097-6 31645541 PMC6811639

[18] Habtemariam S. Berberine pharmacology and the gut microbiota: A hidden therapeutic link. Pharmacol Res (2020) 155:104722. doi: 10.1016/j.phrs.2020.104722 32105754

[19] Hu C Jia W. Diabetes in China: Epidemiology and genetic risk factors and their clinical utility in personalized medication. Diabetes (2018) 67(1):3–11. doi: 10.2337/dbi17-0013 29263166

[20] Mechanick JI Hurley DL Garvey WT. Adiposity-based chronic disease as a new diagnostic term: The American association of clinical endocrinologists and American college of endocrinology position statement. Endocr Pract (2017) 23(3):372–8. doi: 10.4158/EP161688.PS 27967229

[21] Garvey WT Mechanick JI Brett EM Garber AJ Hurley DL Jastreboff AM. A. a. c. e. o. c. p. g. reviewers of the, American association of clinical endocrinologists and American college of endocrinology comprehensive clinical practice guidelines for medical care of patients with obesity. Endocr Pract (2016) 22 Suppl 3:1–203. doi: 10.4158/EP161365.GL 27219496

[22] Pearl RL Wadden TA Tronieri JS Berkowitz RI Chao AM Alamuddin N. Short- and long-term changes in health-related quality of life with weight loss: Results from a randomized controlled trial. Obes (Silver Spring) (2018) 26(6):985–91. doi: 10.1002/oby.22187 PMC5970047 29676530

[23] Gowri V Rizvi SG Squib and A. Al Futaisi S. High-sensitivity c-reactive protein is a marker of obesity and not of polycystic ovary syndrome per se. Fertil Steril (2010) 94(7):2832–4. doi: 10.1016/j.fertnstert.2010.05.007 20561613

[24] Zhou T Heianza Y Chen Y Li X Sun D DiDonato JA. Circulating gut microbiota metabolite trimethylamine n-oxide (TMAO) and changes in bone density in response to weight loss diets: The POUNDS lost trial. Diabetes Care (2019) 42(8):1365–71. doi: 10.2337/dc19-0134 PMC6647048 31332027

[25] Ellulu MS Patimah I Khaza'ai H Rahmat A Abed Y. Obesity and inflammation: The linking mechanism and the complications. Arch Med Sci (2017) 13(4):851–63. doi: 10.5114/aoms.2016.58928 PMC5507106 28721154

[26] Sonnenburg JL Backhed F. Diet-microbiota interactions as moderators of human metabolism. Nature (2016) 535(7610):56–64. doi: 10.1038/nature18846 27383980 PMC5991619

[27] Crovesy L D. Masterson Rosado EL. Profile of the gut microbiota of adults with obesity: A systematic review. Eur J Clin Nutr (2020) 74(9):1251–62. doi: 10.1038/s41430-020-0607-6 32231226

[28] Shin NR Whon TW Bae JW. Proteobacteria: Microbial signature of dysbiosis in gut microbiota. Trends Biotechnol (2015) 33(9):496–503. doi: 10.1016/j.tibtech.2015.06.011 26210164

[29] Li JV Ashrafian H Bueter M Kinross J Sands C le Roux CW. Metabolic surgery profoundly influences gut microbial-host metabolic cross-talk. Gut (2011) 60(9):1214–23. doi: 10.1136/gut.2010.234708 PMC3677150 21572120

[30] Zou Y Chen T. Engineered akkermansia muciniphila: A promising agent against diseases (Review). Exp Ther Med (2020) 20(6):285. doi: 10.3892/etm.2020.9415 33209129 PMC7668130

[31] Chelakkot C Choi Y Kim DK Park HT Ghim J Kwon Y. Akkermansia muciniphila-derived extracellular vesicles influence gut permeability through the regulation of tight junctions. Exp Mol Med (2018) 50(2):e450. doi: 10.1038/emm.2017.282 29472701 PMC5903829

[32] Guo J Han X Zhan J You Y Huang W. Vanillin alleviates high fat diet-induced obesity and improves the gut microbiota composition. Front Microbiol (2018) 9:2733. doi: 10.3389/fmicb.2018.02733 30483238 PMC6243071

[33] Cekanaviciute E Probstel AK Thomann A Runia TF Casaccia P Katz Sand I. Multiple sclerosis-associated changes in the composition and immune functions of spore-forming bacteria. mSystems (2018) 3(6):e00083–18. doi: 10.1128/mSystems.00083-18 PMC6222044 30417113

[34] Cho J Kim D Kang H. Exercise preconditioning attenuates the response to experimental colitis and modifies composition of gut microbiota in wild-type mice. Life (Basel) (2020) 10(9):200. doi: 10.3390/life10090200 32937846 PMC7555193

[35] Cao J Chen M Xu R Guo M. Therapeutic mechanisms of berberine to improve the intestinal barrier function *via* modulating gut microbiota, TLR4/NF-kappa B/MTORC pathway and autophagy in cats. Front Microbiol (2022) 13:961885. doi: 10.3389/fmicb.2022.961885 35935245 PMC9354406

[36] Zhang X Zhao Y Xu J Xue Z Zhang M Pang X. Modulation of gut microbiota by berberine and metformin during the treatment of high-fat diet-induced obesity in rats. Sci Rep (2015) 5:14405. doi: 10.1038/srep14405 26396057 PMC4585776

[37] Wu C Zhao Y Zhang Y Yang Y Su W Yang Y. Gut microbiota specifically mediates the anti-hypercholesterolemic effect of berberine (BBR) and facilitates to predict BBR's cholesterol-decreasing efficacy in patients. J Adv Res (2022) 37:197–208. doi: 10.1016/j.jare.2021.07.011 35499044 PMC9039652

[38] Zhang Y Gu Y Ren H Wang S Zhong H Zhao X. Gut microbiome-related effects of berberine and probiotics on type 2 diabetes (the PREMOTE study). Nat Commun (2020) 11(1):5015. doi: 10.1038/s41467-020-18414-8 33024120 PMC7538905

[39] Wang J Wang L Lou GH Zeng HR Hu J Huang QW. Coptidis rhizoma: A comprehensive review of its traditional uses, botany, phytochemistry, pharmacology and toxicology. Pharm Biol (2019) 57(1):193–225. doi: 10.1080/13880209.2019.1577466 30963783 PMC6461078

[40] Linn YC Lu J Lim LC Sun H Sun J Y. Zhou. Berberine-induced haemolysis revisited: Safety of rhizoma coptidis and cortex phellodendri in chronic haematological diseases. Phytother Res (2012) 26(5):682–6. doi: 10.1002/ptr.3617 22002596

