# Supplementary material for: Clinical efficacy of weight loss herbal intervention therapy and lifestyle modifications on obesity and its association with distinct gut microbiome: A randomized double-blind phase 2 study
Source: Front Endocrinol (Lausanne). 2023 Mar 22;14:1054674. doi: 10.3389/fendo.2023.1054674 (PMC10073537; doi:10.3389/fendo.2023.1054674)
Supplement: Supplementary file 1 [file DataSheet_1.docx]

Tables

Table S1. The percentage of weight loss and BMI reduction greater than 5% in the two groups.

Table S2. Mean changes in analysis of human fat composition in the two group before and after treatment.

Table S3. Mean changes in routine blood and biochemistry index.

Table S4. Adverse reactions during WL-HIT treatment.

Table S5. A summary of the 16s sequencing data. Data are presented as the mean ± SD.

Table S6. The phylotypes were significantly different between the treatment and placebo groups.

Table S1. The percentage of weight loss (and BMI reduction) greater than 5% in the two groups

|  | **Treatment** | **Placebo** | **P value** |
| --- | --- | --- | --- |
| Percentage of participants  that reached 5% body weight loss | 72.22 %  (13 out of 18) | 36.84%  (7 out of 19) | <0.001 |
| Percentage of participants  that reached 5% reduction in BMI | 77.78%  (14 out of 18) | 36.84%  (7 out of 19) | <0.001 |

Note: BMI, Body Mass Index

Table S2. Mean changes in Analysis of human fat composition in the two group before and after treatment.

| Items | Treatment (n=18) | | Placebo (n=19) | |
| --- | --- | --- | --- | --- |
|  | Baseline | Post-treatment | Baseline | Post-treatment |
| Body fat rate (%) | 34.93 ± 8.55 | 31.44 ± 8.3^**##^ | 39.63 ± 4.37 | 38.21 ± 4.57^***^ |
| Body moisture rate (%) | 45.21 ± 3.79 | 44.27 ± 4.06 | 42.79 ± 3.97 | 42.43 ± 4.5 |
| Rate of skeletal muscle (%) | 25.04 ± 1.37 | 26.71 ± 1.66^***^ | 25.31 ± 2.38 | 25.93 ± 2.20 |
| Basal metabolic rate  Kcal/day | 1683.8 ± 271.7 | 1633.5 ± 299.3 | 1599.9 ± 251.7 | 1571.3 ± 258.2 |
| Body Fat (kg) | 34.47 ± 5.65 | 29.37 ± 5.76^***##^ | 37.29 ± 8.58 | 35.59 ± 8.53^***^ |
| Fat-free body weight (kg) | 56.12 ± 16.6 | 54.41 ± 16.53^**^ | 56.72 ± 11.07 | 55.68 ± 11.28^***^ |
| Body fat muscle ratio | 1.47 ± 0.21 | 1.29 ± 0.24^***##^ | 1.58 ± 0.29 | 1.53 ± 0.29^**^ |

Data are shown as mean ± SD. Intra-group analyses were performed by using t test followed by Wilcoxon (*p* value, ^**^< 0.01, ^***^< 0.001), inter-group analyses were performed by using 2-way ANOVA (## represents *p* < 0.01) by Prism 9 software

Body fat (kg) = Body Weight (kg) * Body fat rate (%).

Fat-free body weight (kg) = Body weight - Body fat.

Body fat muscle ratio = Body fat rate (%)/Rate of skeletal muscle (%).

|  |  |
| --- | --- |
|  |  |

**Table S3. Mean changes in Routine Blood and biochemistry index.**

| Items | Normal values | Treatment (n=18) | | Placebo (n=19) | |
| --- | --- | --- | --- | --- | --- |
|  |  | Baseline | Post-treatment | Baseline | Post-treatment |
| WBC (×10^9^/L） | 3.5-9.5 | 6.79 ± 1.61 | 6.6 ± 1.18 | 7.6 ± 1.71 | 7.23 ± 1.39 |
| NE (×10^9^/L） | 40-75 | 4.08 ± 1.16 | 3.96 ± 1.03 | 4.46 ± 1.06 | 4.35 ± 1.04 |
| NE% | 40.0--75.0 | 59.46 ± 6.64 | 57.65 ± 6.79 | 58.54 ± 4.26 | 56.71 ± 5.05 |
| EO% | 0.4--8.0 | 2.30 ± 1.37 | 2.17 ± 1.29 | 1.66 ± 1.13 | 1.72 ± 1.03 |
| RBC（×10^12^/L） | 3.80--5.10 | 4.90 ± 0.37 | 4.94 ± 0.39 | 4.65 ± 0.41 | 4.77 ± 0.39 |
| PLT（×10^9/^L） | 125--350 | 205.61 ± 33.57 | 208.56 ± 34.1 | 220.79 ± 36.01 | 211.79 ± 36.01 |
| HB（g/L） | 115--150 | 145.44 ± 10.05 | 142.83 ± 9.77 | 140.63 ± 12.89 | 136.95 ± 12.71 |
| ALT (U/L) | 0.0--40.0 | 30.44 ± 10.92 | 30.17 ± 8.77 | 32.16 ± 24 | 31.37 ± 28.51 |
| AST (U/L) | 0.0--45.0 | 21.67 ± 4.23 | 21.06 ± 3.69 | 20.63 ± 7.21 | 23.47 ± 10.96 |
| GGT (U/L) | 0.0--45.0 | 42.22 ± 23.57 | 38.65 ± 15.27 | 36.37 ± 24.48 | 39.26 ± 27.85 |
| TBIL (μmol/L) | 5.0--21.0 | 15.31 ± 4.72 | 14.36 ± 3.15 | 11.67 ± 3.56 | 13.03 ± 4.07 |
| TP (μmol/L) | 65.0--85.0 | 73.49 ± 4.54 | 73.44 ± 3.94 | 73.89 ± 4.14 | 75.45 ± 5.95 |
| A (g/L) | 40.0--55.0 | 41.68 ± 2.4 | 42.64 ± 2.39 | 40.95 ± 2.2 | 45.15 ± 5.59 |
| BUN (mmol/L) | 2.5-7.2 | 4.30 ± 1.21 | 4.27 ± 1.09 | 5.03 ± 1.34 | 4.88 ± 0.79 |
| Cre (umol/L) | 45-84 | 61.67 ± 13.22 | 55.83 ± 9.25^*^ | 57.26 ± 12.86 | 57.21 ± 10.89 |

Data was shown as mean ± SD. Intra-group analyses were performed by using t test followed by Wilcoxon by Prism 9 software, *p* value, ^*^< 0.05.

Table S4. Adverse reactions during WL-HIT treatment.

| No. | Time of reaction | Adverse Reactions | Treating methods | Complete treatment or not | Group |
| --- | --- | --- | --- | --- | --- |
| 1 | Three days later | Decreased hyper appetite, bloating | After-meal administration | Yes | Treatment |
|  | A week later. | Mild diarrhea | Light diet, avoid greasy diet, normal the next day |  |  |
| 2 | A week later | Decreased hyper appetite | After-meal administration | Yes |  |
|  | Two weeks later | Eliminates constipation. | No treating |  |  |
| 1 | A week later | Mild nausea, bloating, stomach upset, Decreased hyper appetite | After-meal administration; | Yes | Open trail |
| 2 | Two days later | Nausea, decreased hyper appetite | After-meal administration | Yes |  |
|  | Two weeks later | Increased frequency (3-4 times) of stools for a few days. | Dose reduction by 33%. |  |  |
| 3 | Three days later | A dry and bitter feel in the mouth | No special treatment, normal after two days | Yes |  |
| 4 | Four days later | Nausea, decreased hyper appetite | Disciplinarian bite and sup. | Yes |  |
| 5 | A week later. | Decreased hyper appetite, tired of greasy | After-meal administration | Yes |  |

Table S5. A summary of the 16s sequencing data. Data are presented as the mean ± SD.

| Item | Treatment | | Placebo | |
| --- | --- | --- | --- | --- |
|  | Baseline | Post-treatment | Baseline | Post-treatment |
| Reads | 6001 ± 1281 | 5627 ± 1388 | 6071 ± 1513 | 4907 ± 1632 |
| OTUs | 79.11 ± 31.87 | 80.27 ± 29.11 | 94.81 ± 32.50 | 75.11 ± 23.60 |
| Phylum | 6.16 ± 1.09 | 6.22 ± 1.03 | 5.89 ± 1.15 | 5.89 ± 1.32 |
| Order | 14.88 ± 3.25 | 15.28 ± 3.08 | 15.47 ± 1.98 | 15.11 ± 1.97 |
| Family | 22.62 ± 5.77 | 23.61 ± 4.96 | 24.73 ± 4.32 | 22.47 ± 3.71 |
| Genus | 39.11 ± 10.96 | 40.15 ± 9.86 | 42.15 ± 8.25 | 36.11 ± 6.26 |
| Species | 58.01 ± 19.28 | 59.50 ± 16.85 | 66.52 ± 18.76 | 55.68 ± 13.15 |
| Ace | 93.51 ± 36.76 | 93.91 ± 36.51 | 115.41 ± 31.07 | 93. 09 ± 33.3* |
| Chao 1 | 93.29 ± 38.30 | 94.31 ± 37.10 | 119.28 ± 35.6 | 91.97 ± 31.23* |
| Simpson | 0.23 ± 0.15 | 0.26 ± 0.13 | 0.16 ± 0.13 | 0.19 ± 0.14 |
| Shannon | 2.42 ± 0.69 | 2.37 ± 0.65 | 2.74 ± 0.66 | 2.53 ± 0.55 |

Table S6. The phylotypes were significantly different between the treatment and placebo groups.

| Phylotypes | | T test (Metastats)  TA vs CA | | Treatment  (Relative abundance) | | | Placebo  (Relative abundance) | | |
| --- | --- | --- | --- | --- | --- | --- | --- | --- | --- |
|  |  | p value | Q value | WLTB | WLTA | Riched group | WLCB | WLCA | Riched group |
| Phylum | *Verrucomicrobiota* | 0.004 | 0.043 |  |  | WLTA |  |  |  |
| Species | *Akkermansia_muciniphila* | 0.003 | 0.013 | 4.48% | 10.52% | WLTA | 1.73% | 0.08% |  |
| Phylum | *Firmicutes* | - |  |  |  |  |  |  |  |
| Class | *Clostridia* | <0.001 | 0.005 | 22.17% | 19.65% |  | 33.64% | 37.76% | WLCA |
| Family | *Oscillospiraceae* | 0.014 | 0.054 | 2.45% | 1.83% |  | 2.17% | 0.85% | WLCA |
| Class | *Negativicutes* |  |  | 2.94% | 6.51% | - | 5.72% | 3.13% | - |
| Family | *Ruminococcaceae* | 0.007 | 0.029 | 7.05% | 8.53% |  | 13.91% | 17.23% | WLCA |
| Species | *Megamonas_funiformis* |  |  | 0.03% | 3.14% | - | 0.31% | 0.73% | - |
| Phylum | *Proteobacteria* | - |  |  |  |  |  |  |  |
| Class | *Gammaproteobacteria* |  |  | 32.31% | 29.88% | - | 29.83% | 32.84% | - |
| Genus | *Klebsiella* | 0.003 | 0.019 |  |  |  |  |  | WLCA |
| Species | *Klebsiella_pneumoniae* |  |  | 3.63% | 1.19% |  | 3.07% | 5.42% |  |
| Genus | *Haemophilus* | 0.003 | 0.019 |  |  |  |  |  | WLCA |
| Species | *Haemophilus_parainfluenzae* | <0.001 | 0.005 | 0.15% | 0.00% |  | 0.21% | 1.85% | WLCA |

**Figures**

**
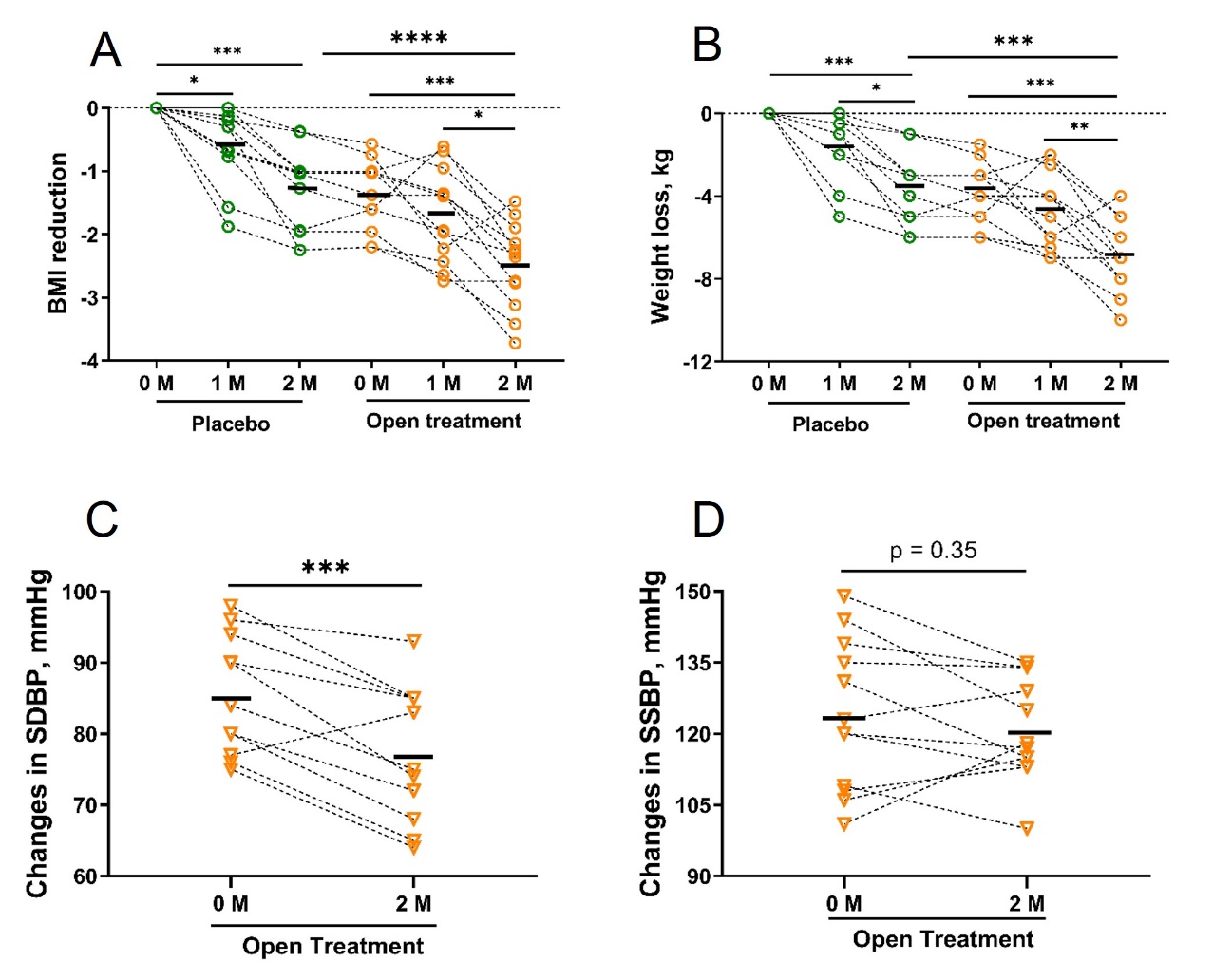
**

**Fig. S1. The reduction in body weight (A) and BMI (B), and mean changes in SDBP(C), and SSBP(D) in secondary measurements.** Bars were shown as mean of each group (n=12). All analyses were performed using one-way ANOVA followed by Bonferroni post hoc by Prism 9 software (*, **, *** and **** represent *p* < 0.05, *p* < 0.01, p < 0.001 and *p* < 0.0001).


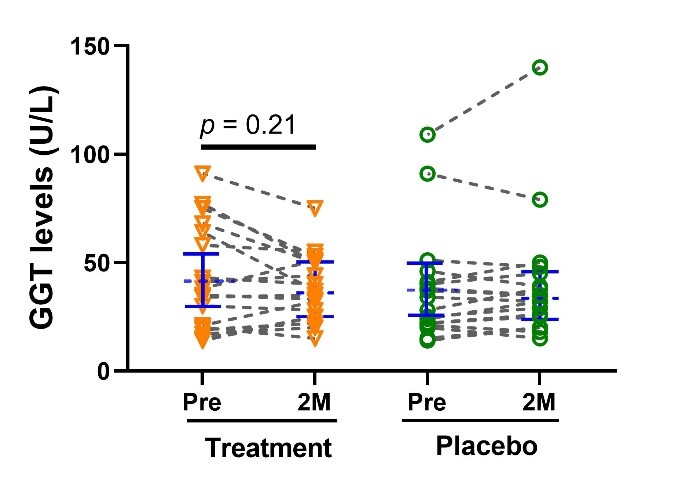


Fig. S2. **Mean Changes in the GGT levels.** Bars were shown as mean of each group with 95% CI. Intra-group analyses were performed using t test followed by Wilcoxon by Prism 9 software (** and *** represent *p* < 0.01 and *p* < 0.001).
